# MUFOLD-DB: a processed protein structure database for protein structure prediction and analysis

**DOI:** 10.1186/1471-2164-15-S11-S2

**Published:** 2014-12-16

**Authors:** Zhiquan He, Chao Zhang, Yang Xu, Shuai Zeng, Jingfen Zhang, Dong Xu

**Affiliations:** 1 Department of Computer Science and C.S. Bond Life Sciences Center, University of Missouri, Columbia, Missouri, 65211, USA

## Abstract

**Background:**

Protein structure data in Protein Data Bank (PDB) are widely used in studies of protein function and evolution and in protein structure prediction. However, there are two main barriers in large-scale usage of PDB data: 1) PDB data are highly redundant in terms of sequence and structure similarity; and 2) many PDB files have issues due to inconsistency of data and standards as well as missing residues, so that automated retrieval and analysis are often difficult.

**Description:**

To address these issues, we have created MUFOLD-DB http://mufold.org/mufolddb.php, a web-based database, to collect and process the weekly PDB files thereby providing users with non-redundant, cleaned and partially-predicted structure data. For each of the non-redundant sequences, we annotate the SCOP domain classification and predict structures of missing regions by loop modelling. In addition, evolutional information, secondary structure, disorder region, and processed three-dimensional structure are computed and visualized to help users better understand the protein.

**Conclusions:**

MUFOLD-DB integrates processed PDB sequence and structure data and multiple computational results, provides a friendly interface for users to retrieve, browse and download these data, and offers several useful functionalities to facilitate users' data operation.

## Background

Protein structure data in Protein Data Bank (PDB) [1] are widely used in studies of protein function and evolution, and they serve as a basis for protein structure prediction. The number of entries in PDB has been increasing rapidly. However, there are two barriers in large-scale usage of PDB data, especially in an automatic fashion. The first barrier is that a large number of protein chains in PDB are highly similar in terms of sequence or structure. For example, many PDB files contain identical chains. Hence, a light version of PDB may be useful. In addition, PDB users often need to obtain a set of PDB chains satisfying some criteria such as structure resolution and sequence length, or they may need to select a representative from a group of similar sequences/structures. The second barrier in large-scale usage of PDB data is that many PDB files have issues due to inconsistency of data and standards as well as missing residues, so that automated retrieval and analysis are often difficult. For example, the sequence in a PDB header is sometimes inconsistent with that in the 3D coordinate part. Another example is that some residues in PDB are modified, and the residue types cannot be easily mapped to the original amino acids. One more issue is that many PDB files have incomplete coordinates containing some residues or atoms without 3D coordinates. This may be due to un-resolved electron density maps. However, it creates problems for a systematic data analysis of large-scale PDB files. Furthermore, if someone likes to perform molecular dynamics simulation or other computational analysis of a given PDB file, it may require preprocessing the file to add coordinates of missing atoms. If the pre-processed PDB files are readily available for download, it may help many simulation users.

Currently, several websites are available to address the first barrier. The PDB website itself can remove similar sequences with specific levels of mutual sequence identity. Other websites such as PDB-Select [2], ASTRAL [3], PDB-REPRDB [4] and PISCES [5] have similar functions, all of which allow users to download a pre-defined chain list or generate a customized list with some sequence or structure criteria. However, the derived chain lists from these websites are typically not updated weekly following the release of hundreds of PDB files each week. Release of non-redundant structure datasets is even slower. For example, the widely used protein structure classification database SCOP [6], which involves extensive manual annotations, was updated years ago (1.75 release in June 2009). It would be useful to incorporate automatic SCOP classification for newly released PDB files, even if the classification quality is suboptimal. In addition, the second barrier in large-scale usage of PDB data, as illustrated above, has not been addressed systematically.

In this paper, we introduce MUFOLD-DB which comprehensively integrates processed PDB data, predicted SCOP classification and additional computational data, e.g. DSSP [7] secondary structure and PSI-BLAST [8] sequence profile. MUFOLD-DB provides a friendly web interface for users to browse, search and download these data. Compared to other databases, MUFOLD-DB has the following unique features:

(1) Users can search a PDB sequence against several derived sequence databases by using BLAST with specified parameters and browse all the hit sequences.

(2) Users can generate a customized list from the entire PDB sequences by setting the filtering parameters, which include full or partial SCOP address, experimental method (e.g., X-Ray or NMR), sequence length, structure resolution (only applied to X-Ray structures), deposit date, and mutual sequence identity level from 90, 80 to 30 percent. This can be used for a non-redundant template database in developing protein energy function and template-based protein structure prediction.

(3) Users can input a list of chain names to browse the corresponding information and quickly get the representatives of the involved clusters after clustering with seven levels of mutual sequence identity, from 90 to 30 percent. This utility can be used to cluster a set of sequences to reduce redundancy.

(4) MUFOLD-DB carefully processes the PDB sequence and structure to provide users clean data which is much easier to manipulate than the original PDB files. Structures of missing regions with less than 7 residues in PDB chains are predicted by high-quality loop modelling using MODELLER [9], to help structure prediction and function analysis.

(5) Multiple data are provided for users to download including sequence, predicted SCOP classification, cleaned PDB format file, and PDB files with loop modelling. Pre-computed sequence and SCOP representative datasets are also provided. These files can be retrieved through a command line without going through a web browser.

(6) Users can view each chain in details. Besides the basic information from PDB files, evolutional information represented as sequence logo, secondary structure, disorder region, and three-dimensional structure visualization with JMol http://www.jmol.org are provided.

(7) The database is automatically updated every week following the weekly release of PDB.

## Construction and content

### Data organization

**Figure 1:**
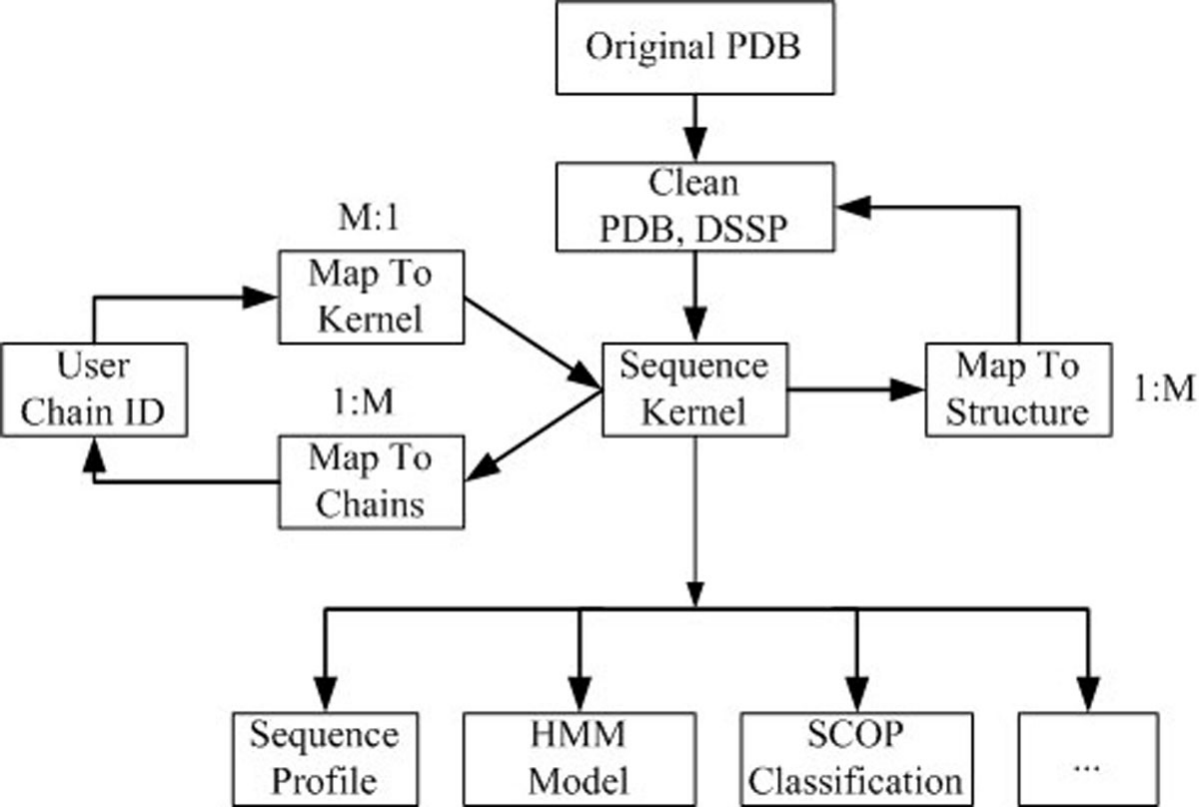
**Data generation and organization**. 1:M (one-to-many mapping); M:1 (many-to-one mapping)

As an automatic routine, MUFOLD-DB weekly synchronizes its PDB files to ftp://ftp.wwpdb.org/pub/pdb/data/structures/all/pdb/ and organizes the processed data in chain units as shown in Figure 1. Original PDB files are processed to have a simplified, clean PDB format. For all cleaned PDB chains, secondary structures are computed using DSSP; and non-redundant sequences (defined as kernel) are generated by mapping multiple sequences to one representative (M:1 or many-to-one mapping). A representative (kernel) sequence or a user-specified chain ID can be mapped to PDB chains through a one-to-many (1:M) mapping by sequence similarity. Sequence profile, hidden Markov Model [10] and predicted SCOP classification are computed for a given kernel sequence. This data organization reduces the data redundancy, and therefore saves storage space and builds up the foundation for various computational purposes.

### PDB sequence and structure processing

Besides the basic information such as experimental method, resolution and deposit date, source and references are retrieved from original PDB files; and the sequence and structure data are processed and cleaned as follows:

1. The sequences from the header (reference sequence) and coordinate part of PDB file for each chain are aligned through sequence alignment in which gap is not allowed in the reference sequence. The alignment shows the sequence positions missing coordinates, some of which are probably disordered regions. Also, residue index for the "ATOM" part is re-ordered starting from one, according to the alignment.

2. We have restored the residue codes for the majority of the modified residues through "MODRES" records from PDB files.

3. Atoms of a residue beyond its standard amino acid composition are simply removed as it is difficult for various structure prediction and analysis tools, e.g., MODELLER [9], to process them.

4. PDB chains are removed if the structures have only CA or sequences with length less than 30 or contain too many unknown residues ('X's).

5. If alternative conformations exist for residues or atoms (e.g., residue 1 of THR in PDB 1CBN has two conformations), only the first conformation is selected.

### Sequence clustering

MUFOLD-DB provides a fast way to generate a subset of chains from the whole dataset with seven levels of sequence identity from 90 to 30 percent. This is implemented by a systematic indexing scheme and pre-computed clustering results. Sequence clustering with threshold from 90 to 40 percent is done using CD-Hit [11]. Clustering into 30 percent of mutual identity is done by all-to-all sequence comparison using PSI-BLAST as the lowest identity cutoff of CD-Hit is 40%. The similarity between two sequences is computed by the PSI-BLAST local alignment identity divided by the average sequence length. The selection of the representative from each cluster is based on combination of sequence length, structure resolution and deposit date. Longer sequence has higher priority to be selected; but if two sequences have a length difference of less than 10 residues, the one with higher resolution will be selected. Here X-ray structures are always assumed more accurate than NMR structures. If these criteria cannot determine the priority, sequences with later deposit date have higher priority as newly resolved structures are more likely to have better quality.

### SCOP classification

We developed an automatic protocol to classify the proteins into SCOP hierarchy. The method of assigning a SCOP address to each protein is as follows:

1. Compare each new sequence in PDB dataset against all sequences of SCOP dataset using PSI-BLAST. Select those hits whose E-value is less than 0.01 and Z-Score of the corresponding CE [12] structure alignment is greater than 4.5.

2. If no hit is found in step 1, compare the query structure to the family representatives of the SCOP dataset. Select those hits whose CE Z-Score is greater than 4.5.

3. When multiple hits are found in step 1 or 2, assign the address of the new protein to the hit with the highest CE Z-Score. When the Z-Score is identical, choose the longest sequence as the representative.

4. Check unassigned regions: If the length is greater than 30 residues, repeat steps 1 to 3 using the sub-sequence; otherwise merge the short unassigned regions to the neighboring domains.

Test results show that this automatic protocol approximates SCOP classification well. We selected 585 proteins from SCOP 1.75 that are not present in SCOP 1.73. Ninety-four of them are multi-domain sequences with 186 domains in total. The remaining 491 proteins have single domain. The test was done against SCOP 1.73. Table 1 shows the assignment accuracy. For multi-domain sequences, if the predicted domain region covers more than 80% of the expected SCOP domain region, the prediction is regarded as correct. The unassigned rate is 4.14% among the 677 (186+491) domains that may represent novel folds. This performance is comparable to the published accuracies for fastSCOP [13], where structures were assigned into SCOP super-families only. Meanwhile, fast-SCOP and other websites, e.g. ASTRAL, are unavailable for weekly updates giving MUFOLD-DB the advantage. When new SCOP sequences are released in the future, MUFOLD-DB classification can be adjusted and updated using the new SCOP classification.

**Table 1:** SCOP classification accuracy.

	Family	Super-family	Fold
Multi-domain (186)	95.16%	96.82%	98.39%
Single domain (491)	92.87%	96.95%	97.35%

### Loop modelling

We developed a protocol to predict the structures of residues with missing coordinates in PDB using the loop modelling functionality of MODELLER as follows:

1. Generate an initial model with the alignment between the sequence in the "ATOM" section and the reference sequence in the "SEQRES" section of the PDB file.

2. Run MODELLER to generate 500 model candidates for residues with missing coordinates (missing loop region).

3. Compute the Root Mean Square Deviation (RMSD) between the model and the original structure, defined as rmsdRest for the structure other than missing loop regions. If rmsdRest is greater than 0.1 Å, remove the model, in order to keep the experimental structure intact.

4. Compute the DOPE [14] energy for each of the remaining models and select the model with the lowest DOPE energy as the final output.

**Figure 2:**
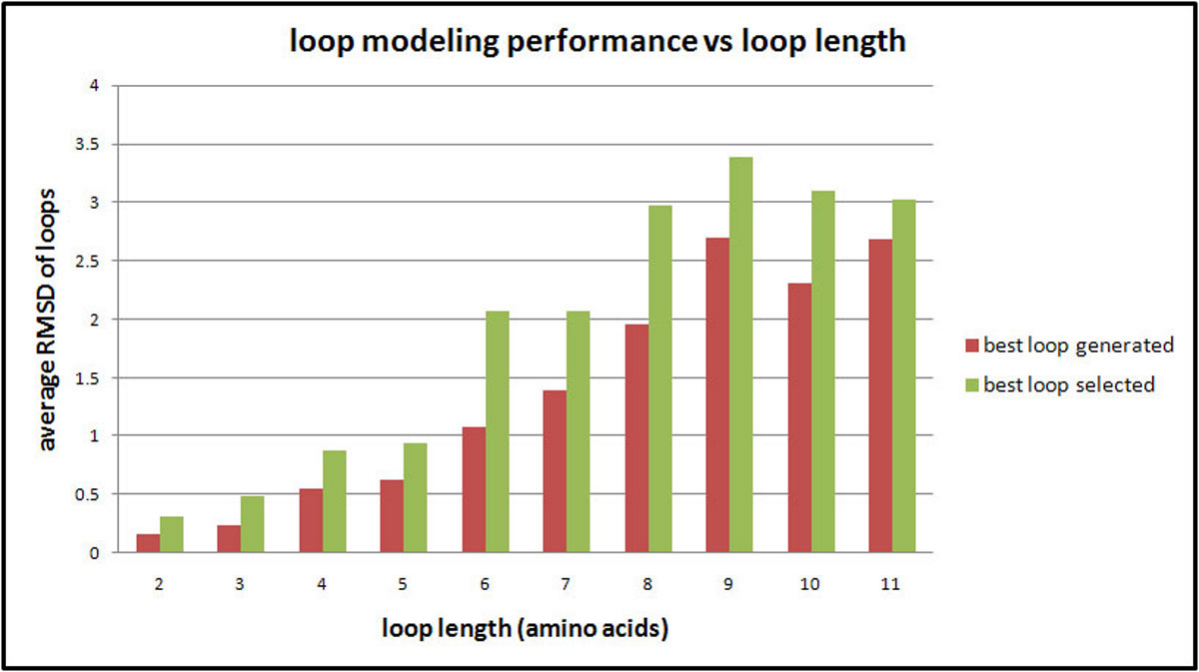
**Loop modelling performance vs. loop length**. X-axis is test sets of different loop length. Each set contains 10 proteins. Y-axis is the average RMSD of loop region between model candidate and original structure.

We tested the above protocol using Modeller 9v3 for 100 randomly selected loop regions, with loop lengths ranging from 2 to 11 residues. We removed the loop regions from the PDB structures and predicted the loop structures. Then we compared the predicted loop structures with the experimental ones as shown in Figure 2. From this figure one can see that when the loop length is shorter than 7 residues, MODELLER can generate very good candidates and the performance of DOPE selection is acceptable. It is expected that some of these loops are in disordered regions, and hence the predicted loop conformations may not be meaningful. However, they are useful in various protein structure prediction and modelling practices (e.g. running a molecular dynamics simulation).

### Additional computational results

To help users better understand the protein, more computational results are integrated. We have calculated sequence profiles for all the sequences by running PSI-BLAST three rounds against the non-redundant (NR) database with the E-value cutoff of 0.001. Sequence profile is represented as a logo image generated using Weblogo [15] for the first 100 alignments extracted from the last round of PSI-BLAST. The secondary structure and solvent accessibility are computed by DSSP. Secondary structure is represented in three states: H (alpha helix), E (beta strand) and C (coil). And relative solvent accessibility (RSA) is computed and classified into three states: E (exposed wherein RSA is greater than 0.37), B (buried wherein RSA is less than 0.069) and I (intermediate, in between) [16]. In addition, a structure image is generated for each chain using Raster3D [17] and MOLSCRIPT [18], and users can view the three-dimensional structure interactively with JMol.

## Utility and discussion

MUFOLD-DB has integrated processed protein sequence and structure data from PDB files and multiple-source information from computational results. It has web-based interfaces and utilities for users to retrieve, browse and download data. The system has some limitations. In particular, the added coordinates for missing residues and atoms are based on computational predictions and may not be reliable. Nevertheless, we believe it provides a valuable resource for the protein modelling community and the structural biology in general.

### Providing a customized list of chains

**Figure 3:**
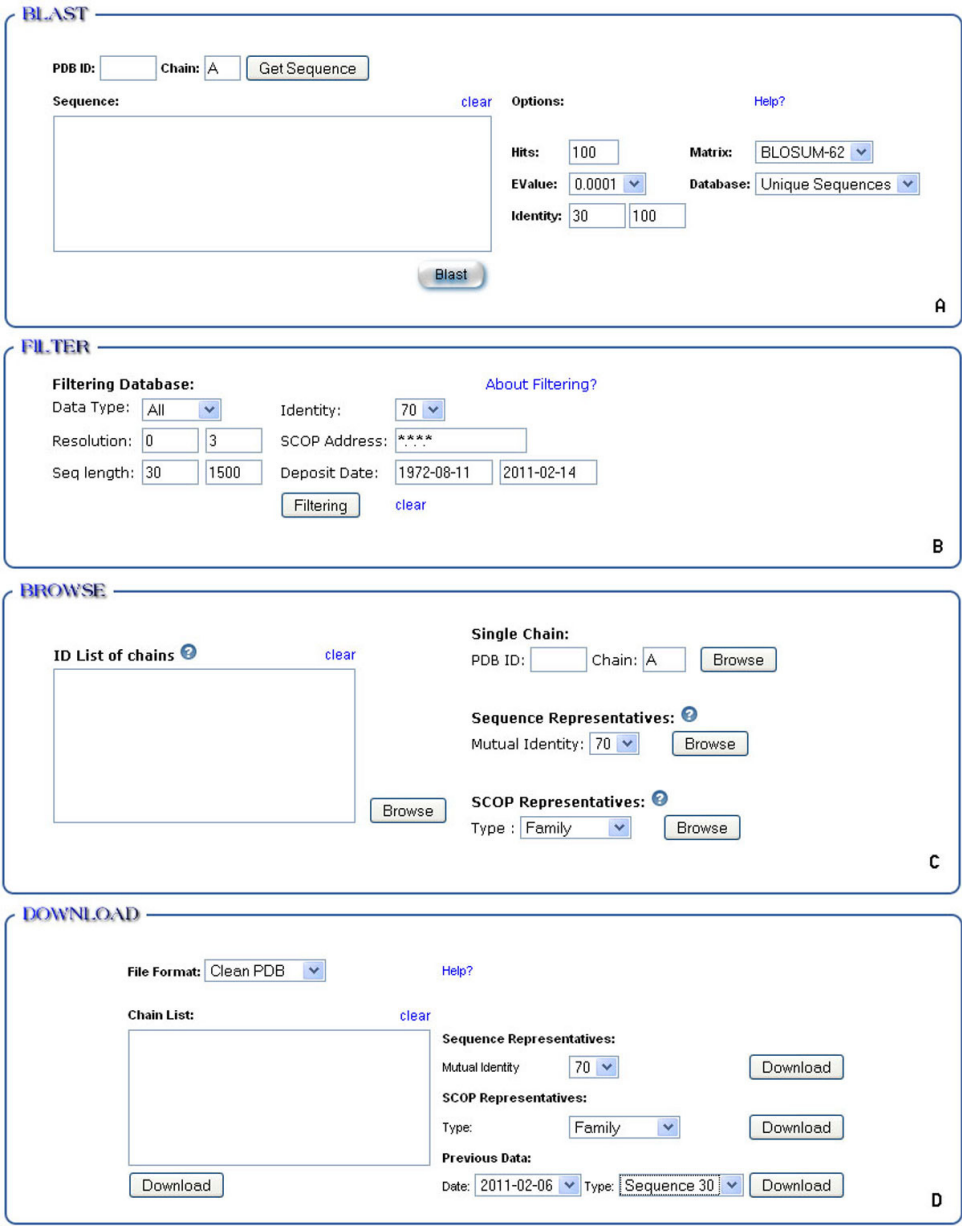
**Web utilities**. These four blocks are copied from the actual web pages. A. Search a sequence against database. B. Get a customized list by setting the filtering parameters. C. Browse a list of chains or the details of a single chain. D. Download data for a list of chains or the pre-computed representative data.

MUFOLD-DB provides two ways to generate a list of chains. One method is to search a database by BLAST (Figure 3A). Users can retrieve a sequence from the database by giving a PDB ID or paste a sequence into the box to search the database using BLAST with different settings. The other method is to filter entire PDB chains by setting the filtering parameters (Figure 3B), e.g. data type, deposit date and SCOP classification. The resultant list of chains is shown pages for users to browse.

### Browse feature provides a list of chains

Figure 3C shows the interface for users to browse the chains on a list generated online or the pre-computed dataset such as sequence representatives of SCOP classification or an input list. The chains will be listed in a table with attributes, ID name, sequence length, structure type, deposit date, source of the protein and predicted SCOP classification. Users can browse and make selections over pages.

### Cluster feature works with input list of chains

Figure 3C has the entry to cluster a set of chains with different levels of mutual similarity.

### Details of single chain available

**Figure 4:**
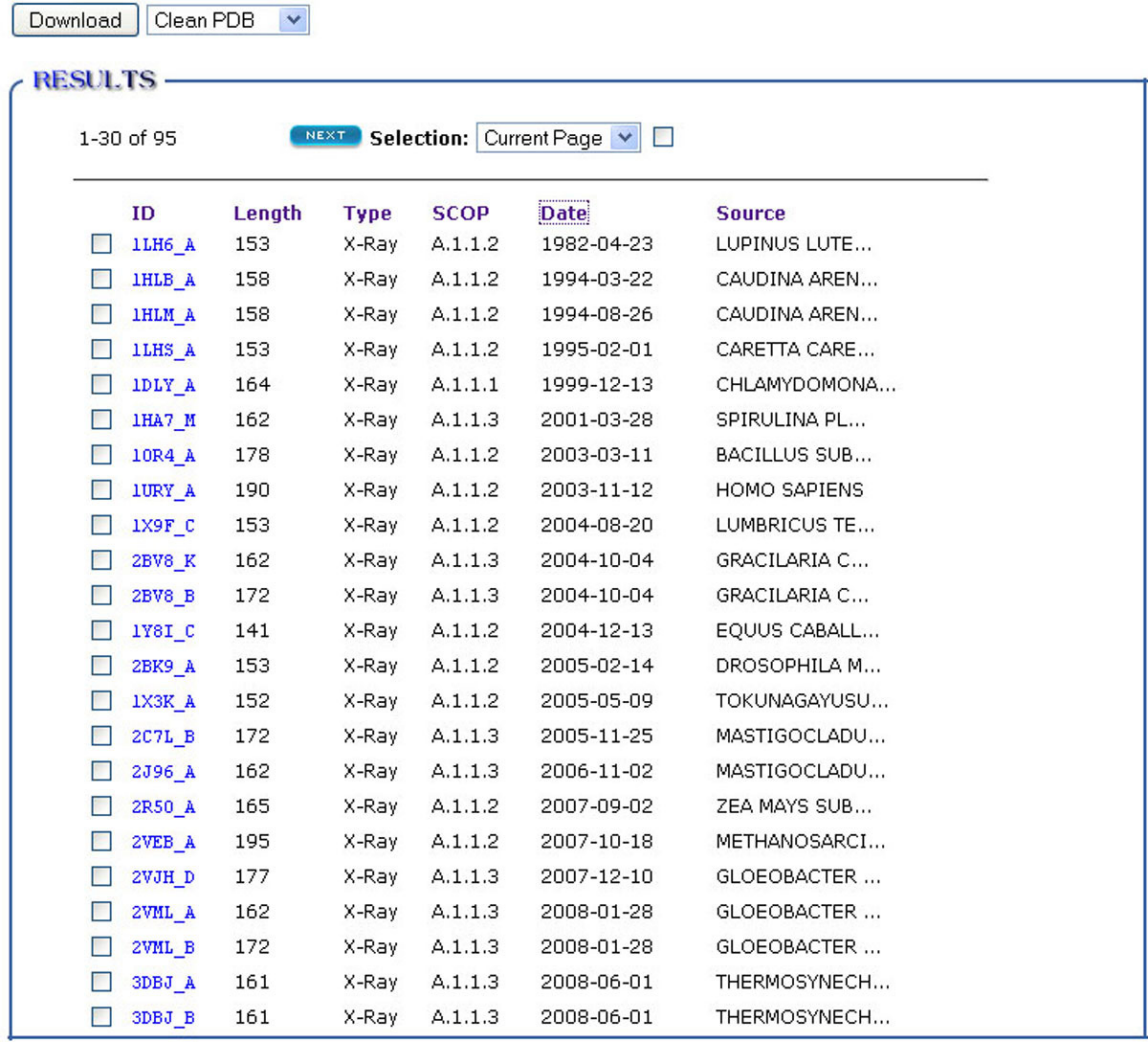
**Listing chains in a table**. This table lists several attributes of each chain, including the experimental data type, released date, predicted SCOP classification and its source information. The table in current page will be sorted if clicking at the corresponding column's name.

**Figure 5:**
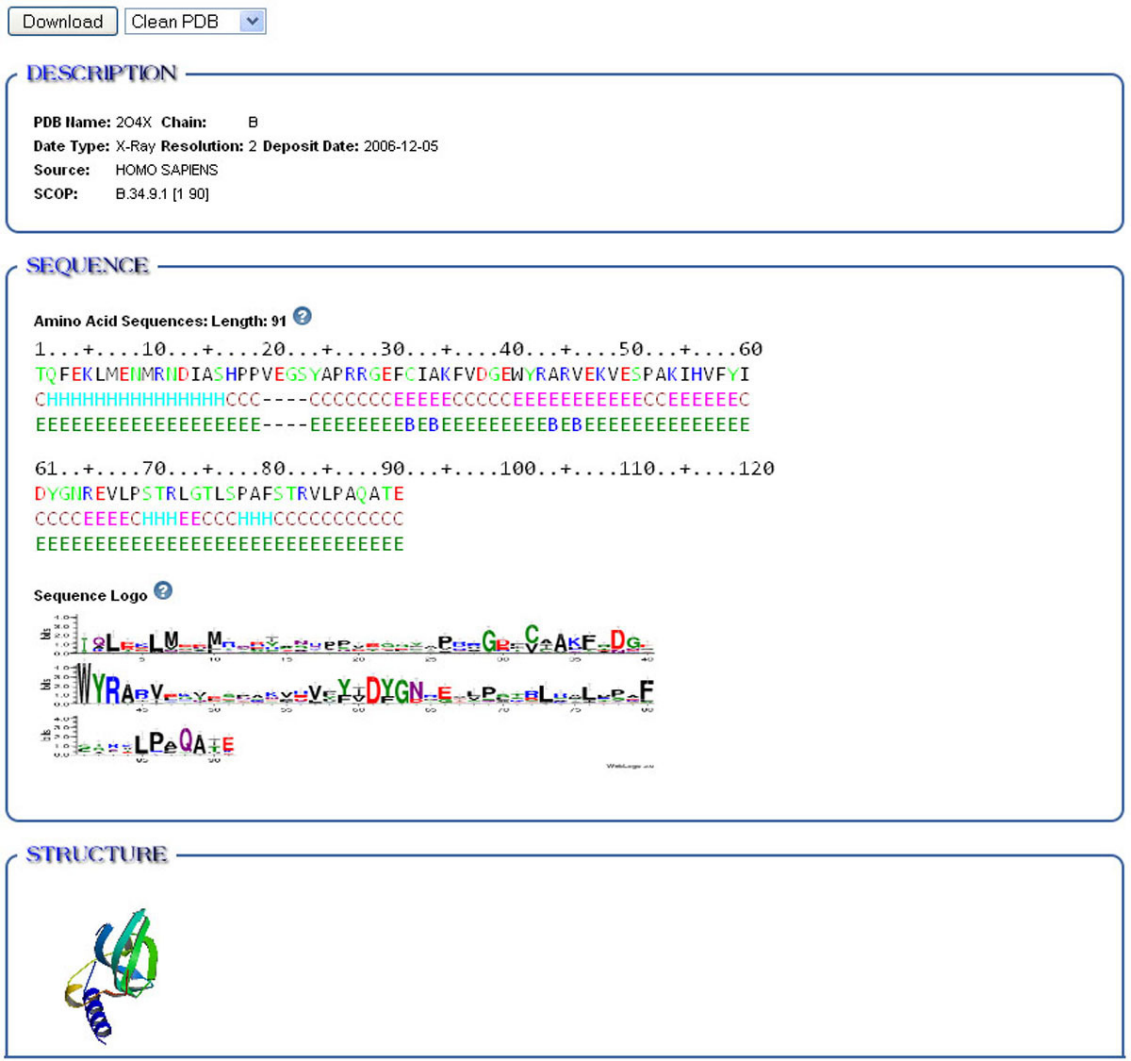
**Detailed information of PDB **2O4X_B. Detailed information of a single chain includes information such as release date, experimental data type, source and computed sequence profile, secondary and 3D structure information.

To see the detailed information of a single chain, users can click the ID in the list page as shown in Figure 4. Figure 3C also has an entry for this. Figure 5 shows the information of chain B of PDB 2O4X. The description part shows it is an X-Ray structure with resolution 2 Å, deposited on December 5, 2006 and its source is *Homo sapiens*. The predicted SCOP address is B.34.9.1. The sequence part shows the amino acid sequence of the chain, secondary structure and regions of missing coordinates. From the sequence logo, users can see the conserved regions in this sequence. Clicking the structure picture will initiate JMol to manipulate the structure.

### Downloading data

Besides the download entry as shown in a result page (see Figure 4), MUFOLD-DB has more options for users to get data. As shown in Figure 3D, users can download the data for an input list, or pre-computed data set, e.g. representative of SCOP classification or sequence clustering with different levels of mutual sequence identity. As MUFOLD-DB is weekly updated, some of its past data are kept. This can be used as a benchmark dataset at different dates.

## Conclusions

As of January 13, 2014, the data of MUFOLD-DB are summarized in Tables 2 and 3. The data cover 2863 SCOP families, 1551 super-families and 960 folds. MUFOLD-DB will be continuously maintained and updated. Part of the future work will be better handling of residue modification and missing coordinates. Currently, we only restore the residue codes and ignore the conformation changes in the atomic coordinates from the modified state to the apo protein. Our study for handling these issues is ongoing and we are also trying new methods to improve the loop modelling performance.

**Table 2:** Number of processed PDB files and the deposit time of the PDB files.

PDB Files	Total Chains	Unique Chains	Deposited From	Deposited To
94,048	243,751	62,649	Aug. 11, 1972	Jan. 13, 2014

**Table 3:** Number of representative sequences at each threshold level of mutual sequence identity.

30	40	50	60	70	80	90
15,953	20,459	23,617	26,163	28,175	30,121	32,815

## Availability

The database is publicly available and can be accessed at http://mufold.org/mufolddb.php

## Competing interests

The authors declare that they have no competing interests.

## Authors' contributions

The MUFOLD-DB is a joint work of the authors as a team. ZH wrote all the scripts and tools to download, process the data and create tables for database. The database and web server were designed and built by CZ and ZH. YX developed and implemented the protocol for SCOP classification and performance analysis. SZ tested and updated the system. JZ and DX provided guidance and critical design decision during the development. All authors contributed to the analyses and discussions. All authors read and approved the final manuscript.
